# Origin of colossal dielectric response in (In + Nb) co-doped TiO_2_ rutile ceramics: a potential electrothermal material

**DOI:** 10.1038/s41598-017-10562-0

**Published:** 2017-08-31

**Authors:** Shanming Ke, Tao Li, Mao Ye, Peng Lin, Wenxiang Yuan, Xierong Zeng, Lang Chen, Haitao Huang

**Affiliations:** 1 Shenzhen Key Laboratory of Special Functional Materials, College of Materials Science and Engineering, Shenzhen University, Shenzhen, 518060 PR China; 2 College of Chemistry and Environmental Engineering, Shenzhen University, Shenzhen, 518060 PR China; 3 Department of Physics, South University of Science and Technology of China, Shenzhen, 518055 PR China; 4 Department of Applied Physics and Materials Research Center, The Hong Kong Polytechnic University, Hung Hom, Kowloon, Hong Kong PR China

## Abstract

(In + Nb) co-doped TiO_2_ (TINO) rutile is an emerging material with a colossal dielectric permittivity (CP) and a low dielectric loss over wide temperature and frequency ranges. The electrical inhomogeneous nature of TINO ceramics is demonstrated by direct local current probing with high-resolution conductive atomic force microscopy (cAFM). The CP response in TINO is found to originate from the electron-pinned defect dipole induced conductive cluster effect and the electrode effect. Two types of dielectric relaxations are simultaneously observed due to these two effects. With the given synthesis condition, we found TINO shows a highly leaky feature that impairs its application as a dielectric material. However, the fast-temperature-rising phenomenon found in this work may open a new door for TINO to be applied as a potential electrothermal material with high efficiency, oxidation-proof, high temperature stability, and energy saving.

## Introduction

Colossal dielectric permittivity (CP) materials have attracted broad attention for the realization of modern electronic devices with miniaturization, integration, and high performance for high energy density storage^1–3^. The *ideal* CP materials should possess simultaneously high temperature- and frequency-stability, CP and sufficiently low dielectric loss. However, the balance between CP, temperature-/frequency-stability and low dielectric loss is still very challenging to be implemented in a single material up to now. The current CP candidates, such as CaCuTi_3_O_12_ (CCTO)^4^, exhibit giant, temperature-independent permittivity (~10^5^) but relatively high dielectric losses (>0.2). Although CP with low loss could be found in ferroelectric or relaxor materials, it is limited within a narrow temperature range close to the phase transition temperature^5^.

(In + Nb) co-doped TiO_2_ (TINO for short) rutile^6, 7^ which has attracted huge attention is recently arising as a *dream* CP material with a colossal permittivity (>10^4^) as well as a low dielectric loss (<0.05) over wide temperature and frequency ranges. Since the discovery of TINO, a series of co-doped rutiles, such as (Sm + Ta),^8^ (Zn + Nb)^9^, (Al + Nb)^10^, (Bi + Nb)^11^, (In + Ta)^12^ and (Ga + Nb)^13^ doped TiO_2_, have been investigated and found to have CP behavior. Liu *et al*.^6^ proposed an electron-pinned defect-dipole model to clarify the CP mechanism of TINO. They suggested that co-doping In^3+^ and Nb^5+^ into rutile produces defect clusters, where the electrons created by Nb^5+^ donor are localized by the presence of In^3+^. These defect complexes formed by co-doping give rise to strong dipoles that are responsible for the extraordinarily high dielectric permittivity. Moreover, the localized electrons lead to extra low dielectric loss. This mechanism describes an intrinsic picture rather than any extrinsic one. However, impedance spectra and I-V results^14–18^ implied that extrinsic effects, such as grain boundary capacitance effect, play an important role in the rutile CP ceramics.

In this work we provide evidence to show that both the contact effect and the electron-pinned defect-dipole play important roles in the CP behavior of (In_0.5_Nb_0.5_)_*x*_Ti_1-*x*_O_2_ (TINO100*x* for short, e.g., TINO10 for *x* = 0.1) ceramics. We performed a crucial experiment on samples with varying contact materials to verify the electrode contribution. We also unambiguously identified the nature of electrical inhomogeneities of TINO ceramics by direct local current probing with high-resolution conductive atomic force microscopy (cAFM).

## Results and Discussion

**Figure 1:**
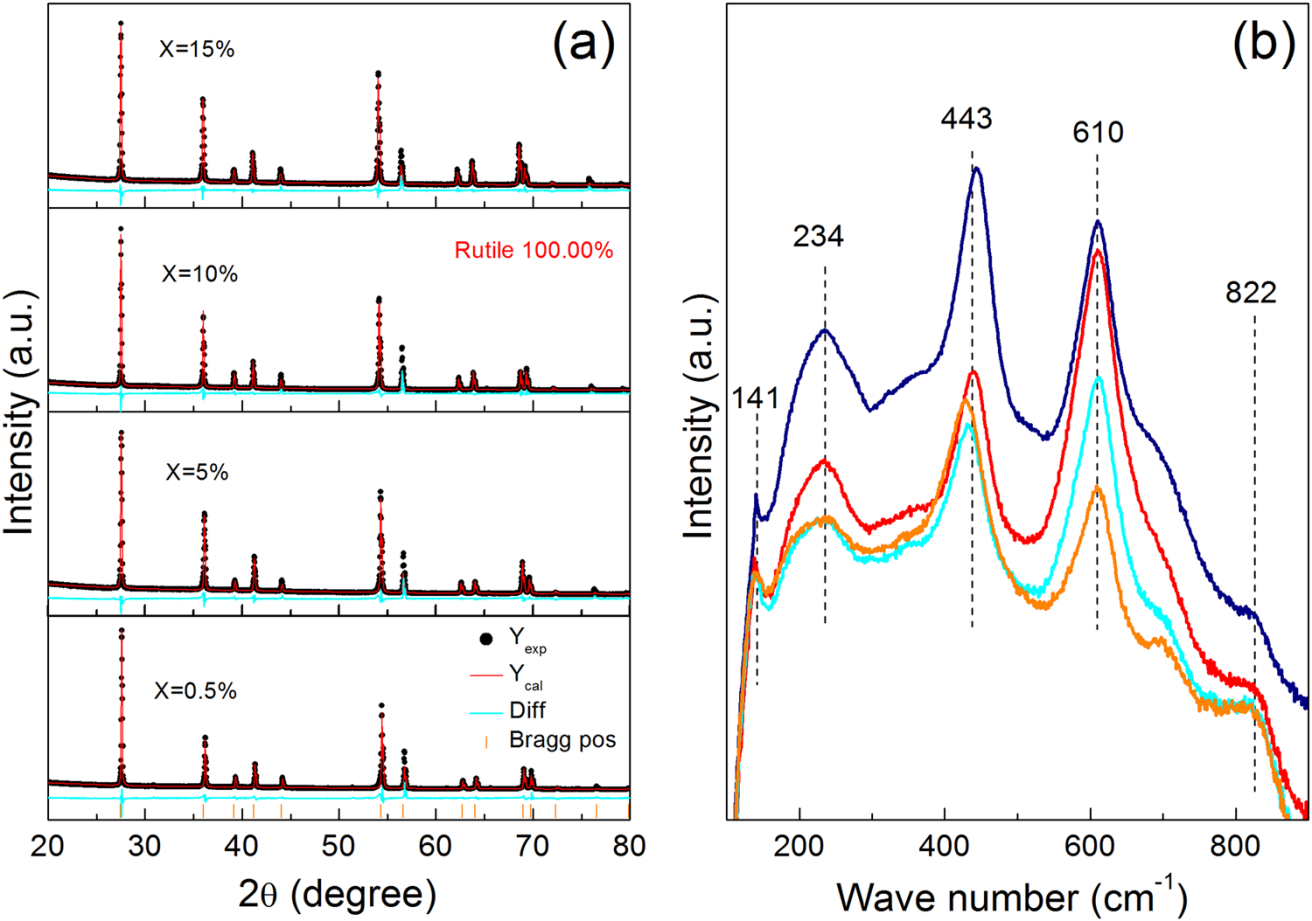
(**a**) XRD patterns of TINO ceramics with different doping concentrations. Rietveld refinement of these XRD results shows that all samples are crystallized in a rutile phase. (**b**) Raman spectra of TINO ceramics.

High-resolution XRD profiles of the sintered TINO samples are shown in Fig. 1a. The results of Rietveld refinement reveal that all of the samples are in pure rutile phase with space group *P4mm*. The lattice parameters and reliable factors are listed in Table S1 (Supporting Information). It shows that the values of *a* and *c* increase linearly with increasing (In + Nb) doping, which is reasonable for larger In^3+^ (radius 0.94 Å) and Nb^5+^ ions (radius 0.78 Å) to replace smaller Ti^4+^ ones (radius 0.74 Å). The Raman spectra shown in Fig. 1b further confirm the pure rutile phase in these samples. Similar to previous results^10, 14^, four Raman active modes are observed: B_1g_ (141 cm^−1^), E_g_ (443 cm^−1^), A_1g_ (610 cm^−1^), and B_2g_ (822 cm^−1^), which are consistent with the standard modes of pure TiO_2_ rutile^19^. The peak around 234 cm^−1^ could be assigned to a multiphonon mode of the second-order Raman scattering in rutile structure^20^.

**Figure 2:**
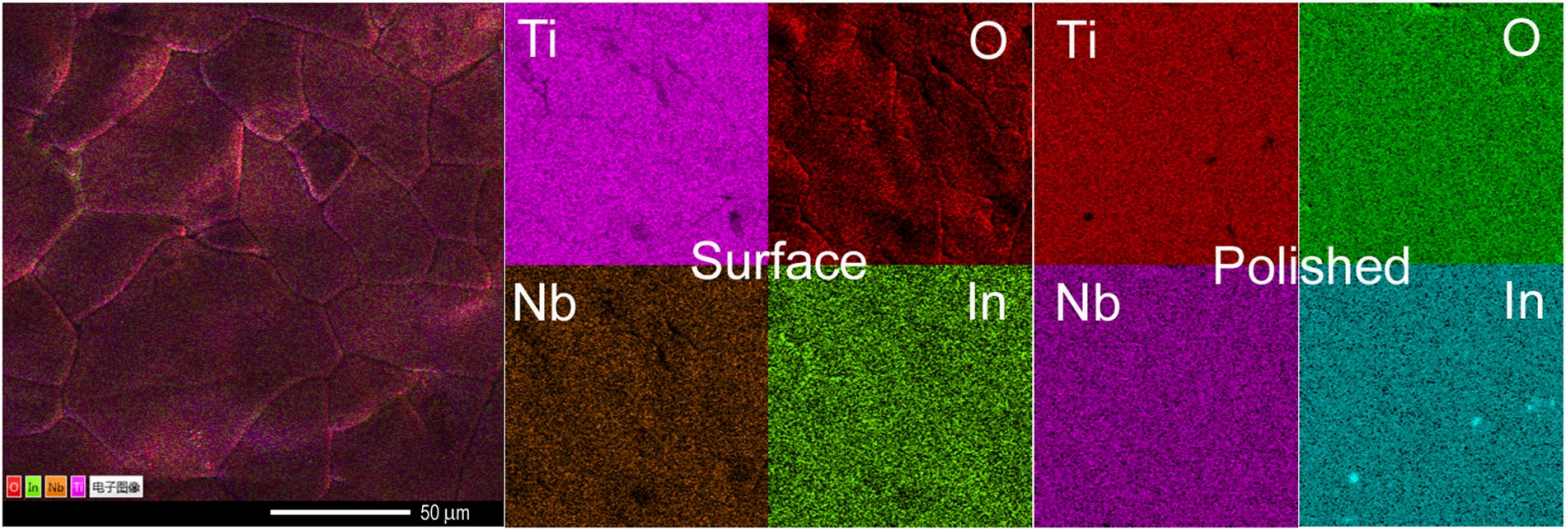
Element mapping of the TINO10 ceramics on fresh and polished surfaces.

Figure 2 shows the SEM images for TINO10 sample sintered at 1400 °C for 24 h, where significantly large grain size (average diameter ~ 40 μm) was observed. The energy-dispersive X-ray spectroscopy of fresh and polished surfaces reveals that the dopants In and Nb are homogeneously distributed in the host matrix, i.e., grains and grain boundaries, which is consistent with previous reports^16^. The other samples displayed quite similar morphology to TINO10 as shown in Figure S1 (Supporting Information), except for a small amount of In segregation on the grain boundaries of the fresh surface. The segregation of In on the grain boundaries of the fresh surfaces suggests the independent existence of Nb^5+^, which did not form defect complex with In^3+^ in TINO. Non-coupled Nb^5+^ induced delocalized electron in the ceramics, leading to the observed electrode effect as shown below and subsequently even high dielectric permittivity and relatively higher loss by comparison to other results^6–18^.

**Figure 3:**
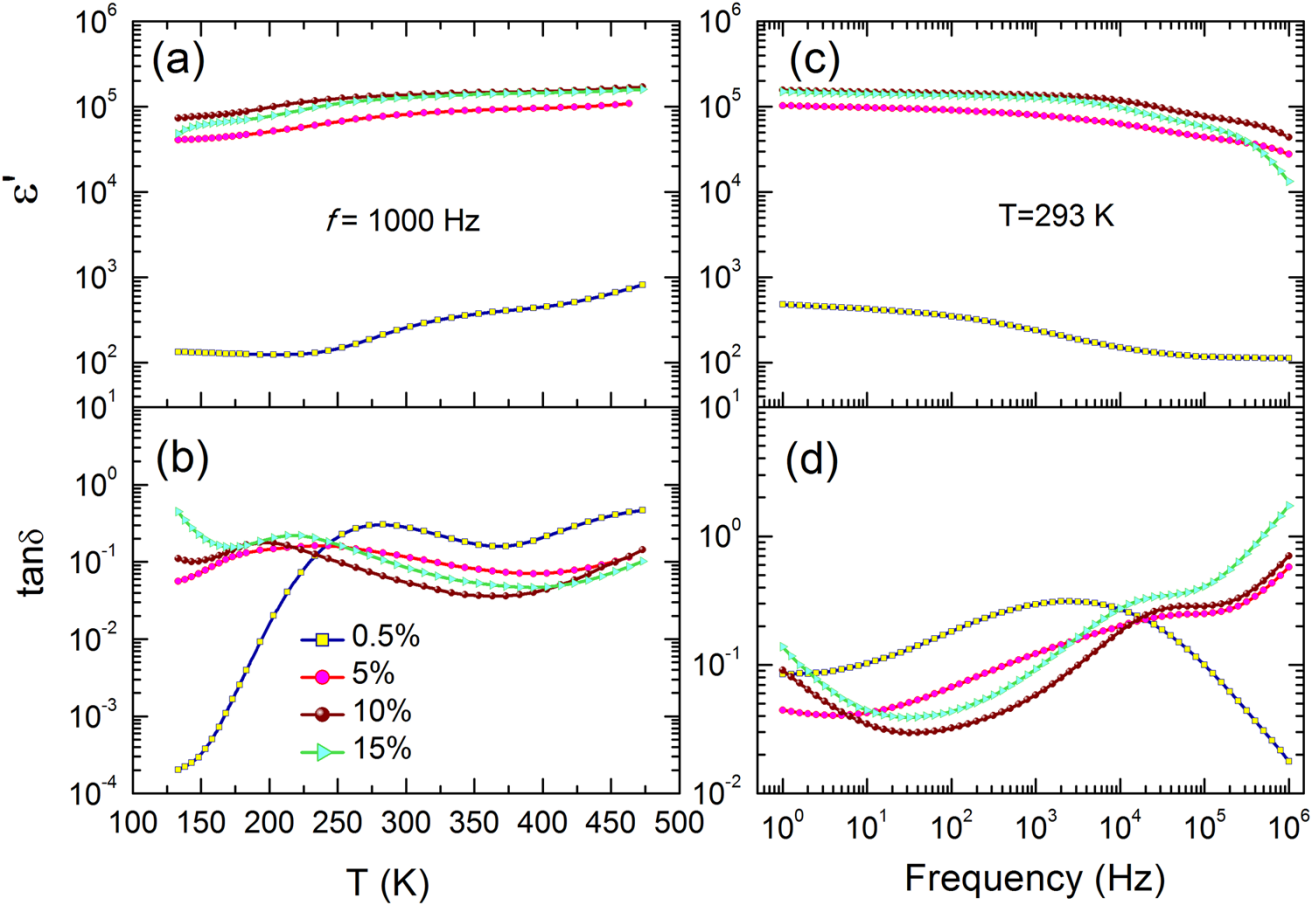
Dielectric response of TINO ceramics with Ag electrodes. (**a**,**b**) Relative dielectric permittivity and loss tangent as a function of temperature at 1000 Hz. (**c**,**d**) Frequency dependent relative dielectric permittivity and loss tangent of TINO ceramics at room temperature.

Figure 3 illustrates the relative dielectric permittivity and dielectric loss as a function of temperature and frequency for sintered TINO samples. Extremely high and nearly frequency-independent dielectric permittivity (1–10^5^ Hz at room temperature) could be observed in TINO with high concentration of (In + Nb) co-doping. For the TINO10 sample, the measured permittivity is as high as ~1.5 × 10^5^ while the dielectric loss is below 0.1 over a wide frequency range. As shown in Fig. 3a, the CP also displays a high temperature-stability over a wide temperature range, from 120 to 500 K. Compared with the work of Liu *et al*.^6^, two differences were observed in this work: (i) The dielectric permittivity decreases with increasing frequency up to 10^6^ Hz. (ii) A dielectric relaxation occurs at around 200 K, where the dielectric permittivity increases from the order of magnitude of 10^4^ to 10^5^. The former is due to the limited response time of the dielectric species (e.g. dipoles) which cannot follow the *ac* electric field under high frequency. The dielectric relaxation of TINO at 200 K was also detected in various other reports^14, 15, 21^. This is primarily due to the interfacial polarization between electrode and the ceramic material, which will be discussed in details next. In addition, it is found that the dielectric permittivity is strongly temperature dependent at a frequency above 100 kHz (Figure S2, Supporting Information), which implies that TINO is probably not suitable for applications in modern high-frequency electronics.

**Figure 4:**
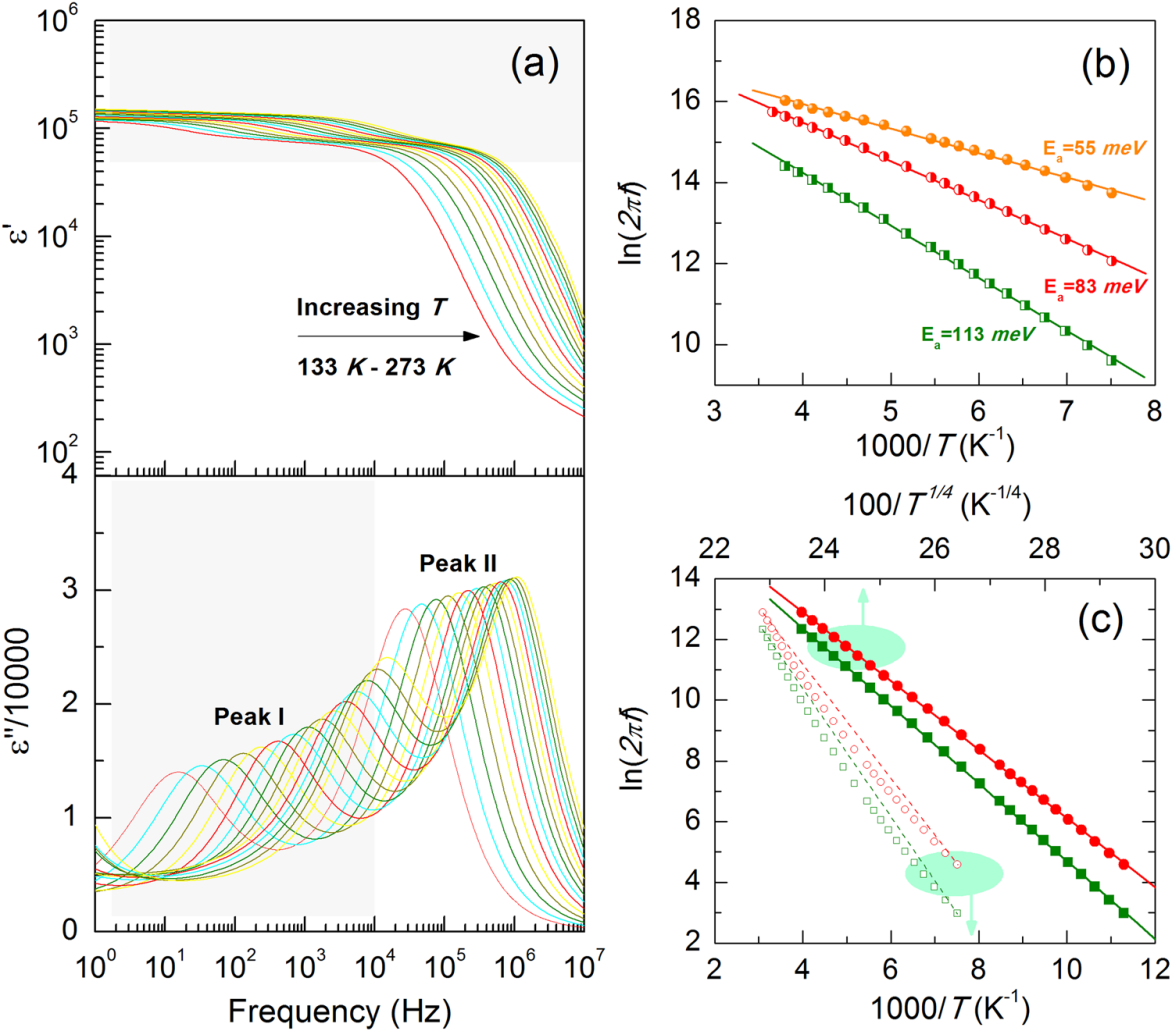
(**a**) Dielectric permittivity of TINO10 as a function of frequency at selected temperatures. Two types of dielectric relaxations can be easily distinguished. (**b**) Arrhenius plots of peak II for TINO5, TINO10, and TINO15. (**c**) Arrhenius and VRH-like plots of peak I for TINO5 and TINO10.

Figure 4a shows frequency dependent relative dielectric permittivity (real part *ε*′ and imaginary part *ε*″) for TINO10 sample at selected temperatures. Similar results could be observed in the *x* = 5% and 15% samples. Two types of dielectric relaxations i.e., peak I for low frequency relaxation (LFR, 1–10^5^ Hz) and peak II for high frequency relaxation (HFR, 10^4^–10^7^ Hz), can be easily distinguished. Both sets of relaxation peaks shift to higher frequencies with increasing temperatures. Clearly, these two dielectric relaxation processes contribute mainly to the apparent CP of TINO ceramics. The HFR process, which is the dominant contribution to the CP reported by Liu *et al*.^6^, accounts for about 50% (≈ 75,000) of the total dielectric permittivity of a TINO10 sample, while LFR contributes the other half.

For a dielectric relaxation, the temperature dependent peak frequency can generally be described by an Arrhenius relation,

1 $$f={f}_{0}\exp (-{E}_{a}/{k}_{B}T)$$

where *E*_*a*_ is the activation energy and *k*_*B*_ is the Boltzmann constant. For HFR (Peak II in Fig. 4a), the data can be well fitted by equation (1), yielding an activation energy of 55 meV for *x* = 5%, 83 meV for *x* = 10% and 113 meV for *x* = 15%. It should be emphasized that a low activation energy (around 10–100 meV) generally indicates thermal behavior of activated electrons/holes, but not the displacement of ions (around 1000 meV, such as ionized oxygen vacancies) and dipoles in oxides^22^. In addition, the LFR process shows quite different behavior (Fig. 4c), where the characteristic frequency deviates from a linear Arrhenius relation but obeys a Mott variable-range-hopping (VRH)-like *T* ^−1/4^ dependence^23, 24^ instead. This result implies an intimate relationship between the low frequency dielectric relaxation and carriers undergoing a VRH movement^25, 26^.

**Figure 5:**
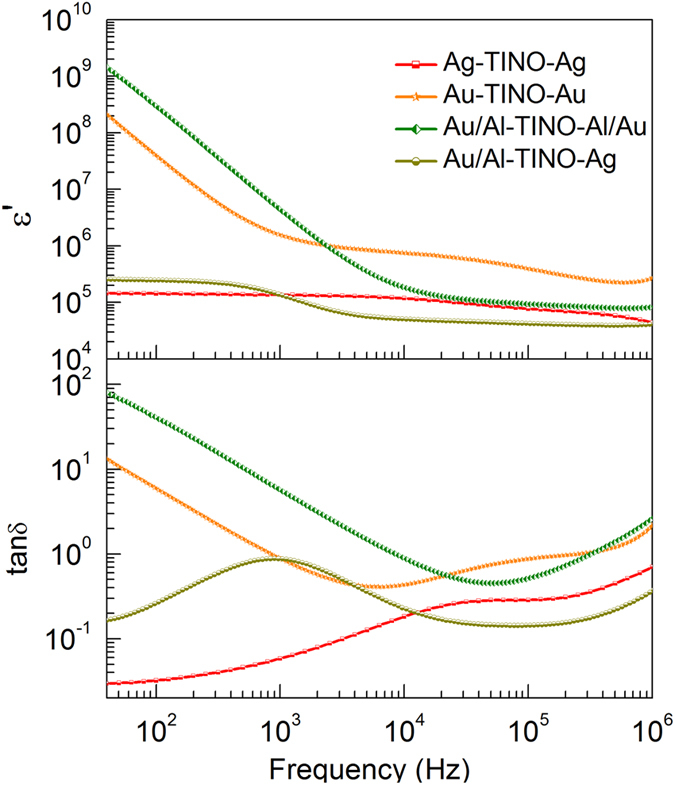
Frequency dependent dielectric permittivity and loss tangent of TINO10 with different electrodes.

**Figure 6:**
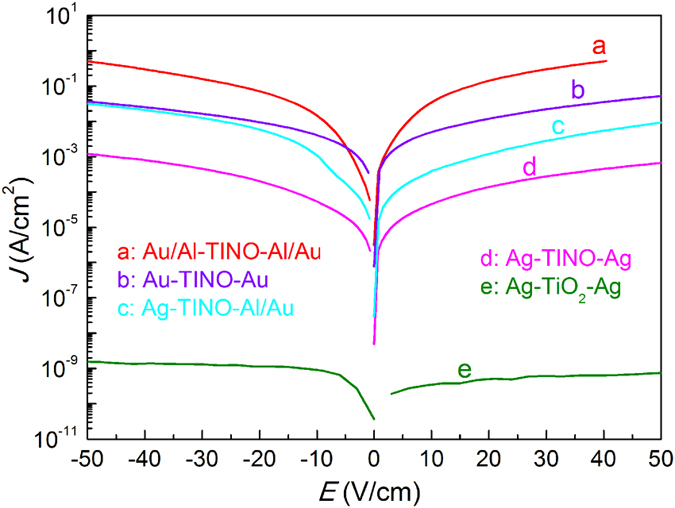
I-V characteristics of TINO10 with different electrodes and a TiO_2_ reference sample. Large leakage current is observed in TINO ceramics.

To further explore the mechanism underlying the observed behavior, we carried out several crucial experiments to study the electrode contributions and internal barrier layer capacitor effect. Different types of electrodes, i.e., Ag, Au and Au/Al, were made. The results of the corresponding frequency dependence, performed at room temperature, are shown in Fig. 5. Depending on the type of electrode, the dielectric permittivity varies considerably. The Au/Al electrode leads to a linear relationship with frequency (below 10^4^ Hz), which is a common feature in materials dominated by *dc* conductivity^27^. The sample with Ag electrode displays a low frequency dielectric relaxation (responsible for LFR) with relatively low dielectric loss. This clearly proves that it’s the contributions from the electrode contact that result in the observed CP and the ceramics are highly semiconducting. The leaky feature of our TINO samples could also be confirmed by their *I-V* curves (Fig. 6). In contrast with pure TiO_2_ ceramics, TINO exhibits unacceptably large leakage current density as a dielectric material. The leakage current density of the Au coated sample is about 0.48 A/cm^2^ under an applied voltage of 5 V (corresponding to 50 V/cm). The current density measured by using Ag electrodes is much lower than the sample using Au/Al electrodes, indicating the formation of a high resistance layer between Ag and TINO. This barrier layer can be further evidenced by analyzing the variation of dielectric permittivity under different *dc* bias fields. In general, a dielectric (non-ferroelectric) material exhibits reduced *ε*′ under a *dc* bias^28^. However, for a leaky capacitor with a depletion layer, the *ε*′ will increase dramatically when the *dc* field overcomes its energy barrier^29^. Figure S3 (Supporting Information) shows such *ε*′*-f* curves under selected *dc* bias fields for TINO10. It is clearly seen that there is a critical bias field, above which the *ε*′ increases significantly at low frequencies. This field is probably comparable to the barrier height between the Ag electrodes and ceramics.

The above results show that the interfacial polarization between the electrode and material is the origin of the low frequency relaxation, which accounts for about 50% of the total CP in TINO (Fig. 4a). This interfacial polarization involves electron movement across a Schottky barrier layer, which follows the Mott’s variable-range-hopping law, leading to the *f* ∝ *T* ^−1/4^ relationship (Fig. 4c). As mentioned above, additional Nb^5+^ exists in our samples without any coupling in defect complex, which supplied delocalized electrons for the interfacial polarization.

The high frequency relaxation (HFR) of TINO gives small activation energy (55–113 meV in this study, as compared with 15 meV reported in ref. 6. It is much smaller than the typical activation energy (~1000 meV) of ions, oxygen vacancies and/or dipoles in oxides. For instance, the activation energy of oxygen vacancies is 910 meV for BaTiO_3_^22^, 1005–1560 meV for KNbO_3_^30^, and 1030 meV for YBCO ceramics^31^. Liu *et al*. attributed this low activation energy to the activation/freezing of electrons in the defect-dipoles^6^. Similar behavior has also been found in perovskite ceramics with an energy of 10–20 meV for the activation of polaron-like defects^23, 26^. It is noteworthy that these electron-pinned defect-dipoles behave “intrinsically” within grains rather than at grain boundaries. However, based on the study of *I-V* behavior on a single grain and grain boundary of TINO ceramics, Li *et al*. referred this HFR process to the grain boundary capacitor effect^14, 16^. Although different in detailed mechanisms, both the electron-pinned defect dipole effect and the grain boundary capacitor effect imply an electrically inhomogeneous structure in TINO ceramics. In order to characterize the detailed inhomogeneous structure, the conductivity mapping of TINO was conducted by high-resolution local current probing with cAFM.

**Figure 7:**
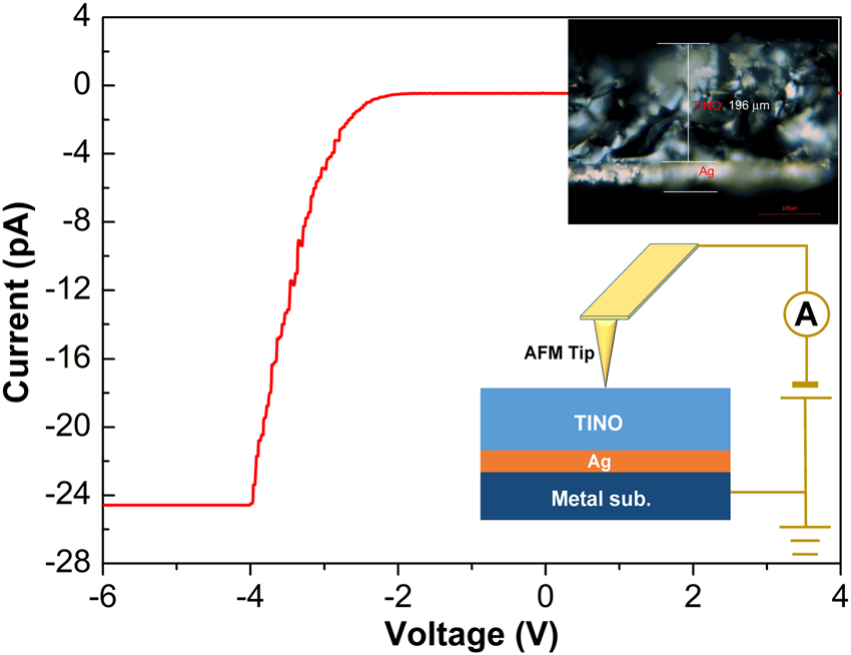
I-V characteristic of polished TINO10 by using cAFM. The inset shows a schematic diagram of the cAFM measurement set up and a cross-section image of TINO10 sample.

**Figure 8:**
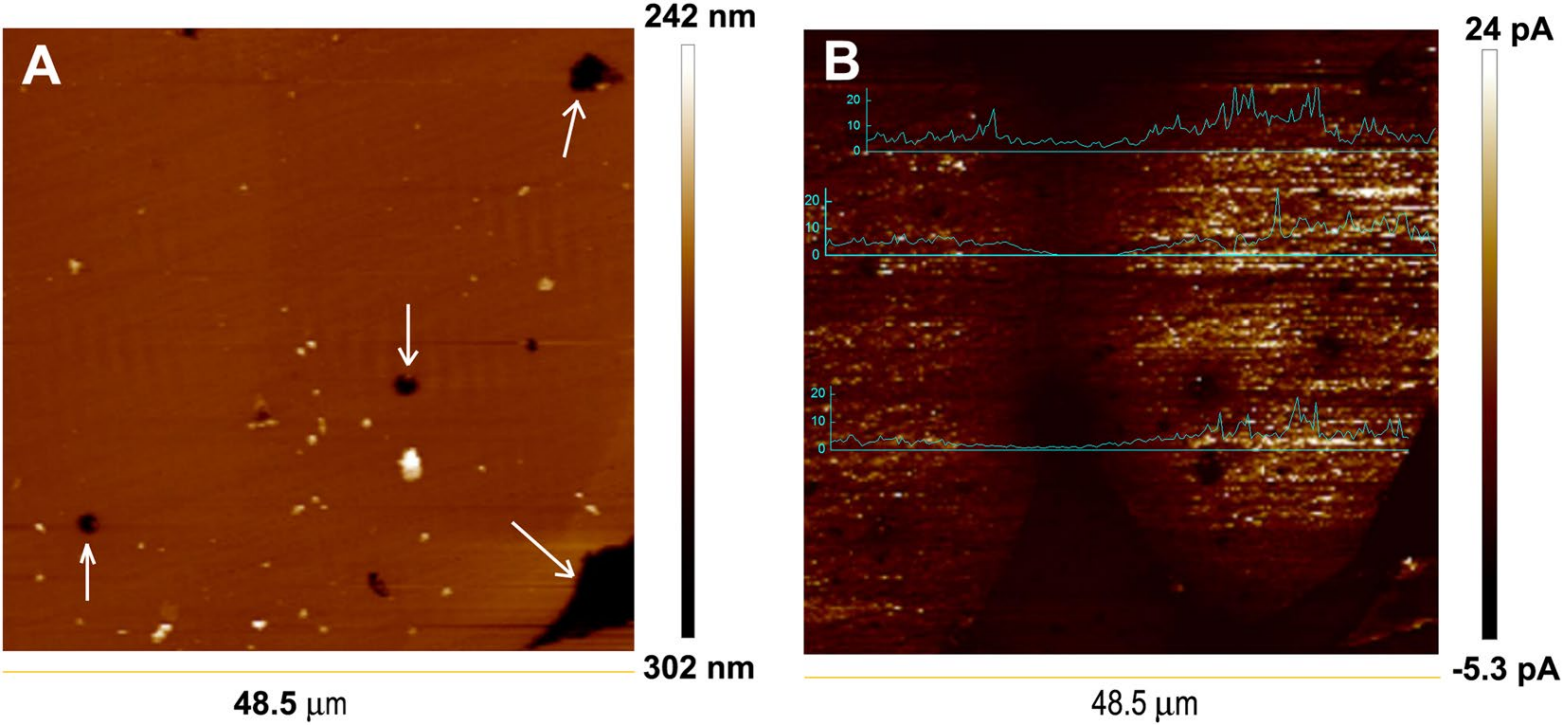
(**a**) Topographic image and (**b**) the corresponding current mapping of the polished surface of TINO10 ceramic sample. Conductive clusters are visible in grains.

A schematic diagram of the cAFM measurement set up is shown in the inset of Fig. 7 with a cross-section image of TINO. The thickness of the measured sample is about 196 μm. The current-voltage (*I-V*) at one grain site displays a strong nonlinear behavior, indicating that the charge transfer is blocked by inhomogeneous areas. Figure 8 shows the AFM topography and AFM current image mapping under a bias voltage of −5 V on a polished TINO10. A negative bias between AFM tip and bottom electrode was used to prevent the block of current due to the interface barrier between the tip and sample surface. In Fig. 8b, the bright contrast represents a conducting state, while the dark contrast represents an insulating state. Although the topography (Fig. 8a) of the polished surface cannot tell the difference between grains and grain boundaries, the current image clearly shows conducting grains and insulating grain boundaries. Figures S4 and S5 (Supporting Information) illustrate the AFM topographic and current image mapping for another area of the same sample. A close examination shows that the electrically inhomogeneous structure consisting of semiconducting and insulating regions within the same TINO grain. The current images provide a direct clue on the location of the conducting clusters. The conducting clusters within the grains are likely due to the defect clusters proposed by Liu *et al*.^6^. According to the X-ray photoelectron spectroscopy (XPS) results (Figure S6, Supporting Information), the oxidation state of Nb in TINO is + 5 and that of In is +3. Noticeable Ti^3+^ signals were also detected. It is well known that the doping of In^3+^ acceptor in TiO_2_ requires oxygen vacancies for charge compensation, while Nb^5+^ doping induces a shallow donor impurity energy level at 0.03 eV^32^, accompanying the reduction of Ti^4+^. The electrons in such shallow donor energy levels are probably localized by $2I{n}^{3+}-{V}_{O}^{\cdot \cdot }$ defect clusters to form local conductive areas as shown in Fig. 8b and Figures S4 and S5. This implies that these localized electron hopping clusters together are responsible for the observed HFR dielectric relaxation. The above speculation could be further evidenced by comparing the dielectric responses of Nb^5+^ and In^3+^-only doped TiO_2_. Nb^5+^-Ti^3+^(Ti^4+^ + e) are formed in Nb^5+^-only doped TiO_2_, where the electrons are virtually delocalized^33^ and can thus hop freely to give high low-frequency dielectric loss (as shown in Figure S7). On the other hand, $2I{n}^{3+}-{V}_{O}^{\cdot \cdot }$ defects would only slightly alter the dielectric permittivity of In^3+^-only doped TiO_2_ from those of pure TiO_2_. Figure S8 shows the AFM topographic and AFM current image mapping of Nb^5+^-only doped TiO_2_ sample. No conducting cluster can be clearly observed. The conducting clusters could be only found in co-doped TiO_2_, which indicates an electron-pinned effect. It should be also emphasized here that electrons localized in TINO by other sources, such as nanoscale structural disorders^34^ and domain boundaries^35^, cannot be excluded by our experiments.

The above results clearly indicate that the colossal dielectric response in TINO is essentially due to the contact effect and inhomogeneous conduction channels within the grain. Our cAFM data provide a direct evidence of the existence of electron-pinned defect clusters, which account for the CP behavior and low dielectric loss in TINO. Furthermore, the electron-pinned defect clusters could be tuned by electrical field and then display an interesting bipolar resistive switching behavior in the thin film form. Figure S9 illustrates such behavior of a TINO thin film sample with a thickness of 150 nm. Two different resistive states could be clearly observed, similar to the case discussed in ref. 36. The low and high resistive states show very good retention performance, indicating a promising application in resistive random access memory. Besides the pinned electrons, it is also worth noting that the synthesis procedure should be controlled very carefully to ensure the fully forming of defect clusters. Since In is a highly volatile element, non-coupled Nb^5+^ exists easily and is prone to leading to delocalized electrons and then higher dielectric loss. Considering the previous work on oxide ceramics with volatile element^28, 37^, a slight of excess In in raw materials and/or sintering under In-rich atmosphere may be helpful to suppress the amount of non-coupled Nb^5+^. Figure S10 displays the dielectric loss results of TINO10 ceramics with different amount of excess In element and sintering under In-rich atmosphere. It demonstrates that the dielectric loss could be reduced greatly in a wide frequency range.

**Figure 9:**
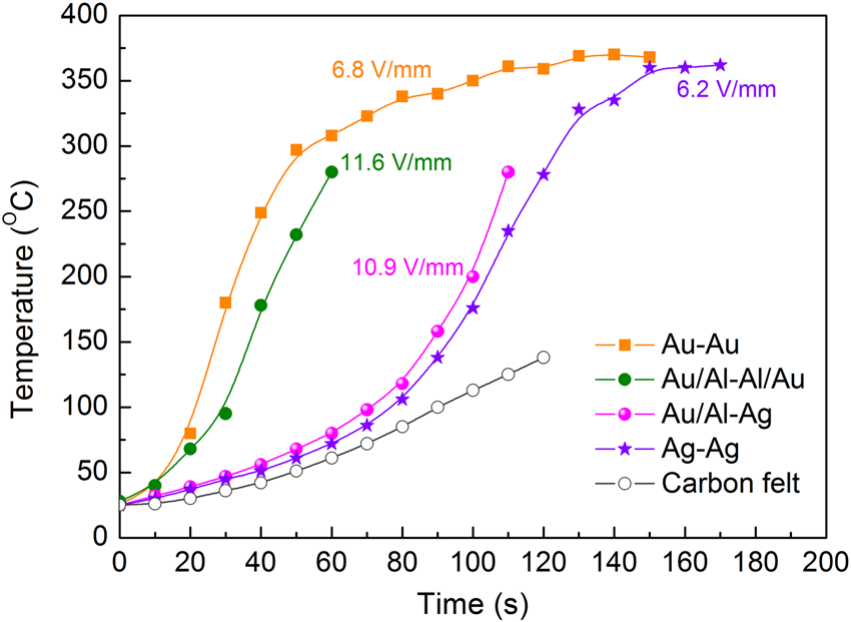
Electrothermal effect in TINO10 with different electrodes.

The highly leaky features of TINO ceramics cast doubts on its applications as a dielectric material with the given synthesis condition. However, an interesting fast-temperature-rising phenomenon was observed in TINO ceramics under a *small dc* bias field (~6 V/mm), which opens a new door for TINO to be applied as a potential electrothermal material with high efficiency, oxidation-proof, high temperature stability, and energy saving. Figure 9 illustrates the time-dependent temperature variation of TINO10 surfaces with different electrode configurations. The surface temperature rose to above 300 °C within 50 seconds under 6.8 V/mm for the Au/TINO/Au structure. If the electric field was increased to 20 V/mm, 400 °C could be achieved in a few seconds, which is 2–6 orders of magnitude faster than common electrothermal materials (e.g. carbon felt, SiC, Al_2_O_3_, etc.). In addition, TINO oxide ceramics display obviously better oxidation-proof and wearable features compared to the carbon-based electrothermal materials. It could be potentially used in various heaters with high heating speed.

## Conclusions

In summary, this work clarifies the origin of colossal dielectric response of (In + Nb) co-doped TiO_2_ ceramics with the given synthesis condition. We provide evidence that the electrode effect and the electron-pinned defect dipoles play important roles in the CP behavior of TINO. The dielectric permittivity could reach to 1.5×10^5^ due to these two effects, while the interfacial polarization led to relatively higher dielectric loss. The electrically inhomogeneous nature was observed clearly by conductive AFM in a large scale. The results shed doubts on the dielectric applications of this dream CP material, but offer another route to use TINO as a potential electrothermal material.

## Methods

### Sample preparation

The raw materials are TiO_2_ (99.99%, Aladdin), Nb_2_O_5_ (99.95%, Aladdin), In_2_O_3_ (99.99%, Aladdin), C_16_H_36_O_4_Ti (99.9%, Aladdin), Nb(OEt)_5_ (99.95%, Sigma-Aldrich), InN_3_O_9_∙xH_2_O (99.9%, Aladdin) without any purification. (In_0.5_Nb_0.5_)_*x*_Ti_1-*x*_O_2_ (TINO, *x* = 0–15%), In_*x*_Ti_1-*x*_O_2_ (TIO, *x* = 10%) and Nb_*x*_Ti_1-*x*_O_2_ (TNO, *x* = 10%) ceramics were prepared by a conventional solid-state reaction method under an In-rich atmosphere to prevent In volatilization. The optimized sintering conditions for these ceramics are 1400 °C for 24 h with a heating rate of 2 °C min^−1^. TINO10 thin films were prepared by a standard sol-gel spin-coating method as described in ref. 38.

### Structure characterizations

X-ray diffraction patterns of the sintered samples were collected on a Bruker D8 Advance SS/18 kW diffractometer with the Cu Kα radiation. Accurate lattice parameters were got by the Rietveld refinement method with Topas 3.1 software^39^. The phase purity was also confirmed by a Raman spectroscopy system (Invia Reflex, Renishaw). Microstructure and element distribution were determined by field-emission scanning electron microscopy (Nova NanoSEM 450, FEI). The valence states were studied by XPS on a MICROLAB 350 apparatus (Thermo Scientific).

### Electrical and dielectric measurements

The dielectric properties were measured by a frequency response analyzer (Novocontrol Alpha-analyzer) over a broad frequency range at various temperatures. The Ag paint electrodes were coated on the samples and fired at 650 °C for 20 min. Au electrodes (~100 nm in thickness) were sputtered on the sample surface after removing the silver paint in an ultrasonic bath and polishing. Au/Al electrodes were made by consecutive sputtering of Al (~60 nm) and Au (~100 nm) after removing the Au electrodes by polishing. In AFM measurements, the system of Bruker Dimension Icon with nanoelectrical application module (PeakForce TUNA) was used; whose current amplifier provides access to the full fA to μA current range. A conductive doped-diamond coated AFM tip with 150 nm nominal radius was scanned over the surface of polished TINO ceramic using Bruker’s PeakForce tapping mode. The DC *I-V* curves were measured by a semiconductor characterization system (2400, Keithley) connected to the probe station. For electrothermal effect measurement, the sample temperature was monitored by a thermocouple attached to the sample surface when a dc bias field was supplied by Keithley 2400.

## Electronic supplementary material

Supporting Information
