# Supplementary material for: Origin of colossal dielectric response in (In + Nb) co-doped TiO2 rutile ceramics: a potential electrothermal material
Source: Sci Rep. 2017 Aug 31;7:10144. doi: 10.1038/s41598-017-10562-0 (PMC5579281; doi:10.1038/s41598-017-10562-0)
Supplement: Supplementary file 1 — Supporting Information [file 41598_2017_10562_MOESM1_ESM.pdf]

Supporting Information

Origin of colossal dielectric response in (In+Nb) co-doped TiO_2 rutile ceramics: a potential electrothermal material

Shanming Ke,[1] Tao Li,[1] Mao Ye,[1] Peng Lin,[1] Wenxiang Yuan,[2] Xierong Zeng,[1] Lang Chen,[3] and Haitao Huang[4]

[1] Shenzhen Key Laboratory of Special Functional Materials, College of Materials Science and Engineering, Shenzhen University, Shenzhen 518060, PR China

[2] College of Chemistry and Environmental Engineering, Shenzhen University, Shenzhen, 518060, PR China.

[3] Department of Physics, South University of Science and Technology of China, Shenzhen, 518055, PR China

[4] Department of Applied Physics and Materials Research Center, The Hong Kong Polytechnic University, Hung Hom, Kowloon, Hong Kong, PR China.

Correspondence and requests for materials should be addressed to W.Y. (email: wxyuanster@gmail.com) or S.K. (email: smke@szu.edu.cn)

Table S1. The Lattice parameters *a*, *c* and *V*, and reliable factors for TINO ceramics obstained by Rietveld refinement.

Compound	X=0%	X=0.5%	X=5%	X=10%	X=15%
Crystal Structure	Rutile	Rutile	Rutile	Rutile	Rutile
a or b (Å)	4.56594	4.59743	4.60604	4.61969	4.63008
c (Å)	2.94489	2.96458	2.97249	2.98614	2.99372
V (Å^3)	61.51	62.66	63.06	63.73	64.178
T (°C)	30	30	30	30	30
Space group	P4mm	P4mm	P4mm	P4mm	P4mm
R-bragg (%)	5.01	6.14	4.14	5.28	3.55
Rwp (%)	8.42	11.29	7.39	8.51	6.71
Rp (%)	5.23	7.18	4.68	5.27	4.30

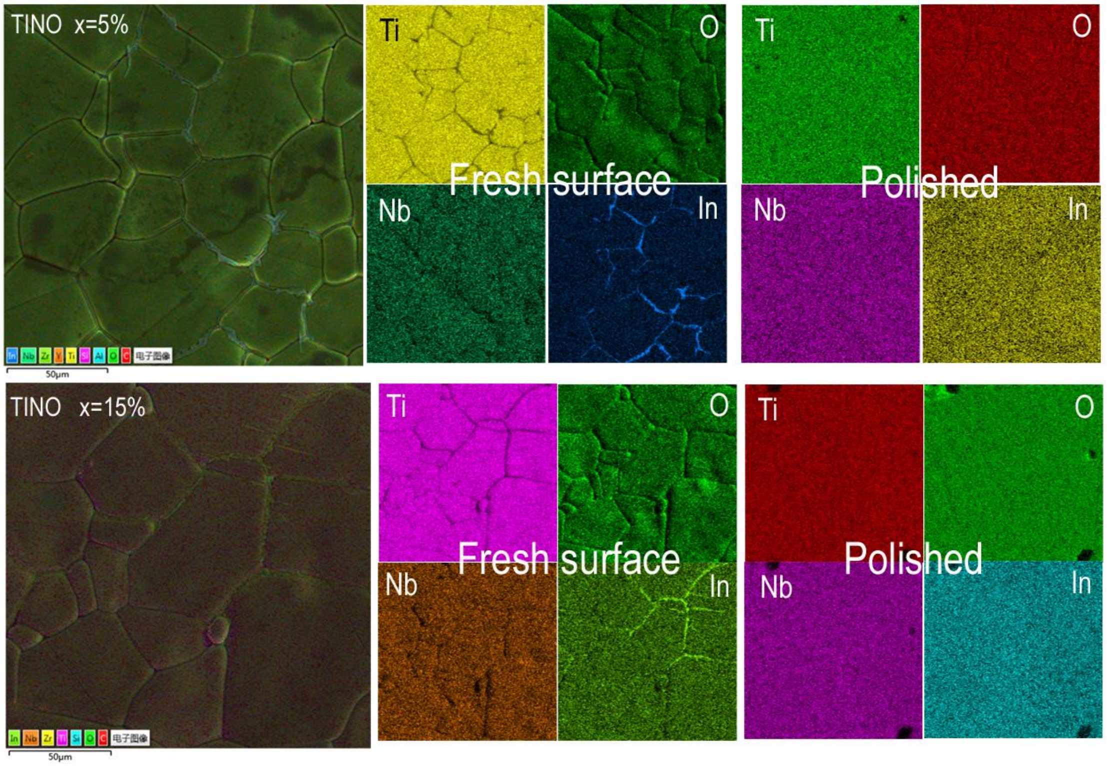

Figure S1. Element mapping of the ceramics with x=5% and 15% across fresh and polished surfaces.

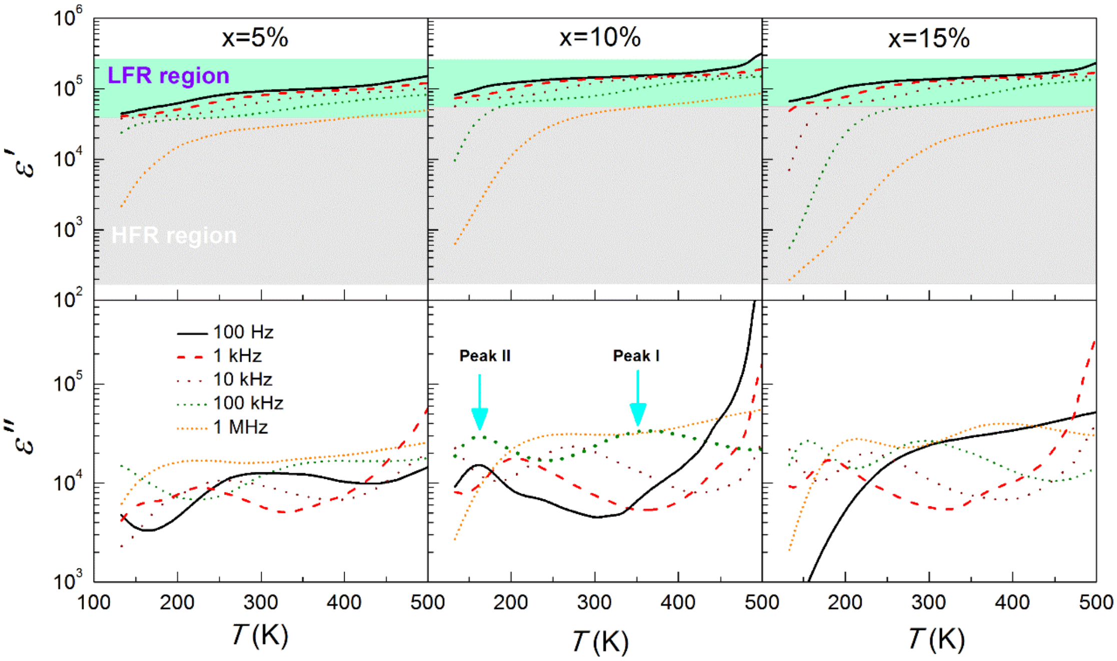

Figure S2. Dielectric constant of TINO5, TINO10, and TINO15 as a function of temperature at selected frequencies.

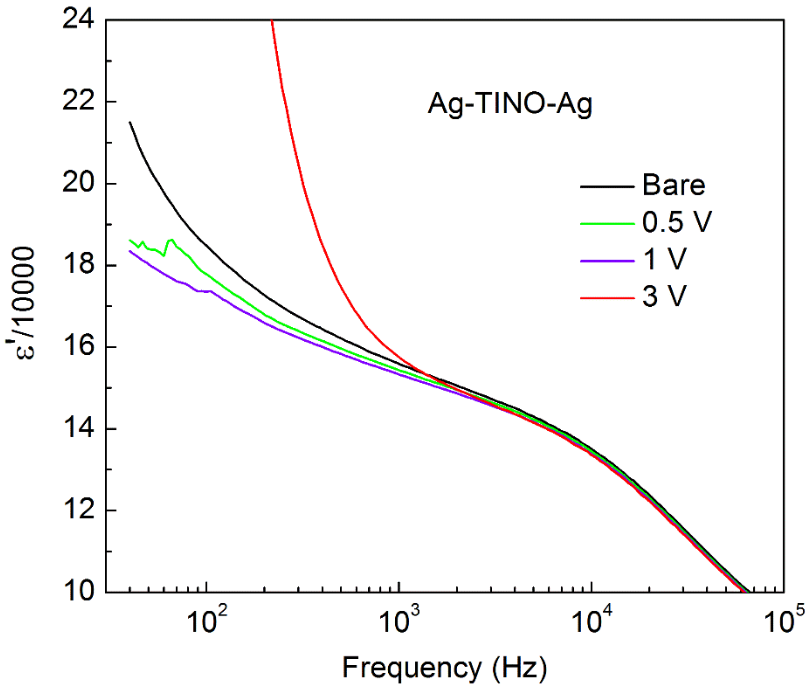

Figure S3. Frequency dependent of dielectric constant of TINO10 under different bias field.

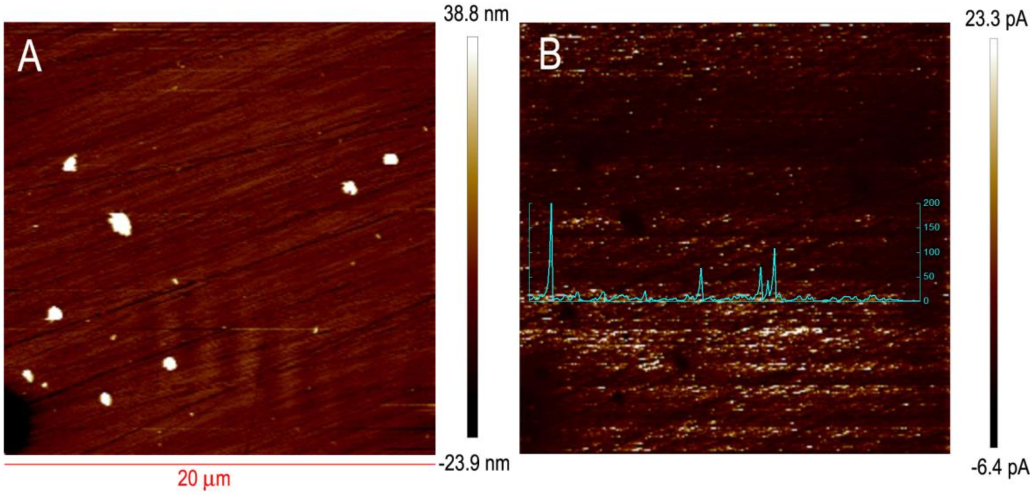

Figure S4. Topographic (a) and current mapping (b) images obtained from polished surface of TINO10 ceramic sample simultaneously. (selected area)

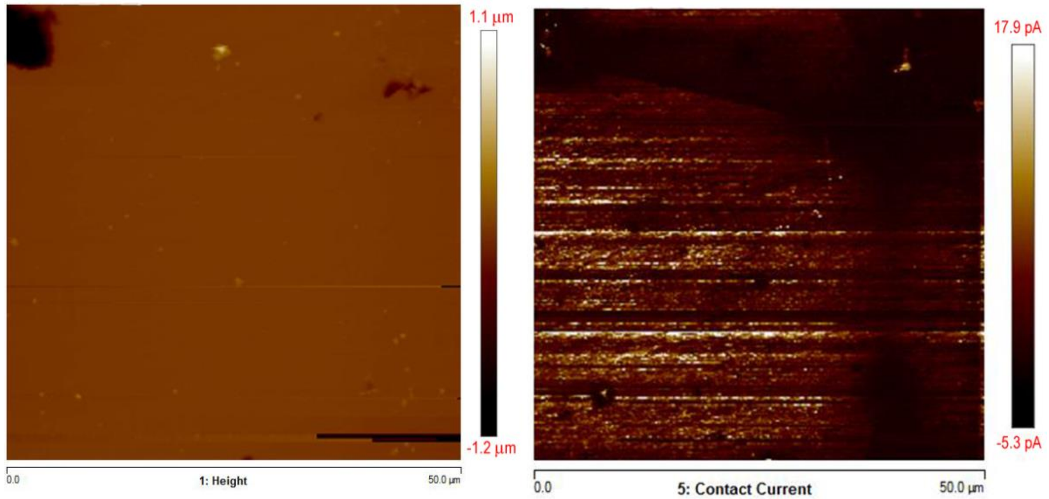

Figure S5. Topographic (a) and current mapping (b) images obtained from polished surface of TINO10 ceramic sample simultaneously. (selected area)

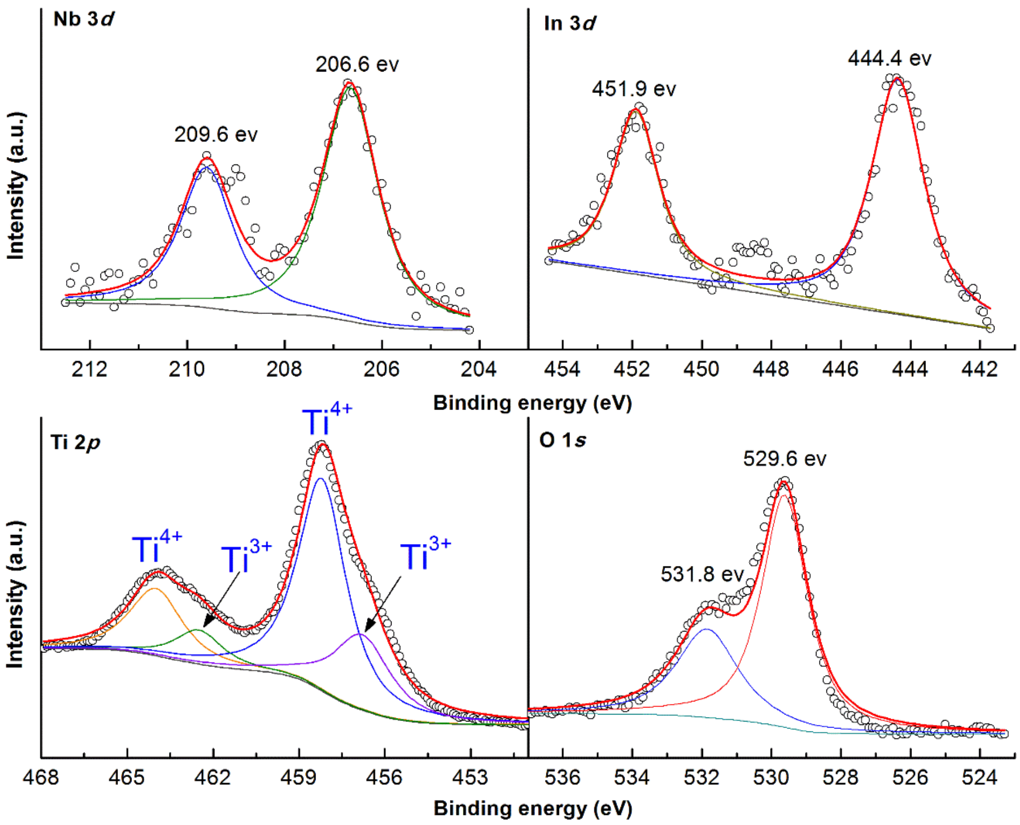

Figure S6. Valence states of the elements in TINO10. The solid lines are fitting curves which consistent with the peaks.

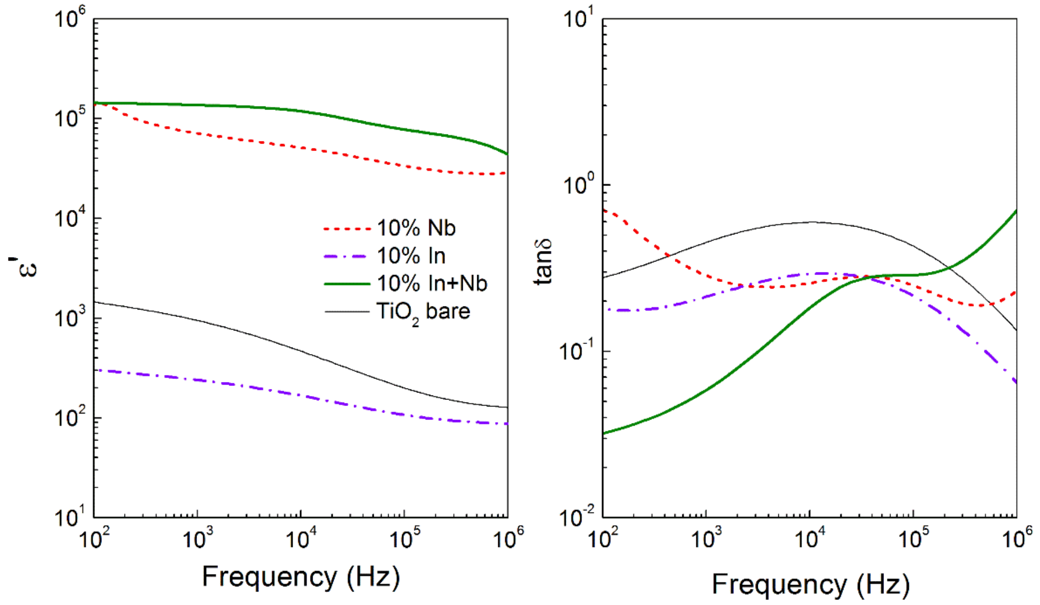

Figure S7. Frequency dependent of dielectric constant and loss tangent of pure, In-doped, Nb-doped, and (In+Nb) co-doped TiO_2 ceramics at room temperature.

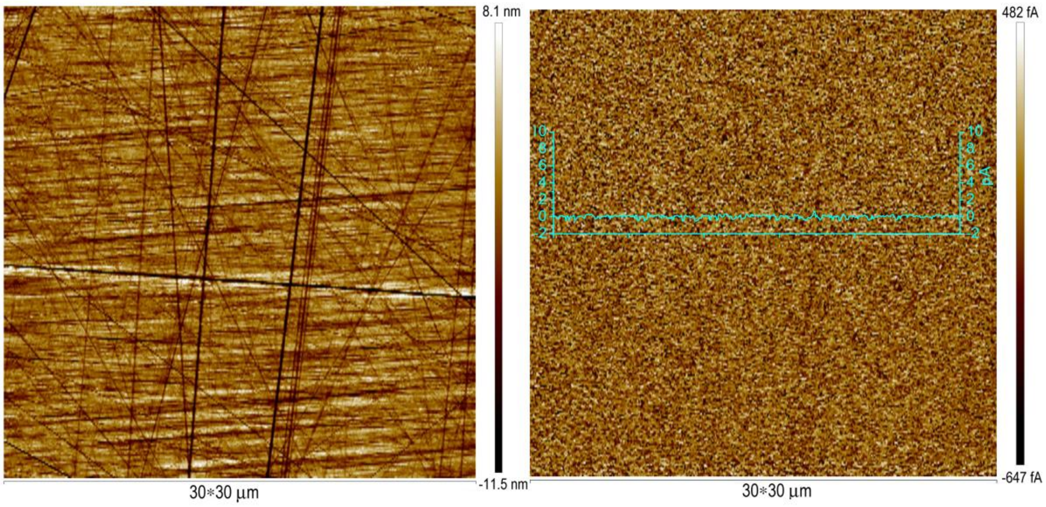

Figure S8. Topographic (a) and current mapping (b) images obtained from polished surface of 10% Nb doped TiO_2 ceramic sample simultaneously. No conductive clusters could be observed.

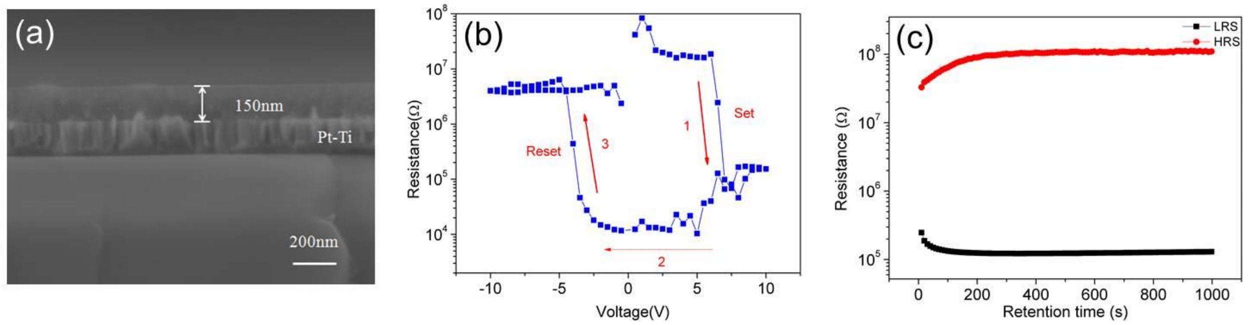

Figure S9. (a) Cross-section SEM image of TINO thin film prepared by sol-gel method. (b) Resistance state of TINO thin film under the scan voltage from 0 V→ 10 V → 0 V → -10 V → 0 V. Two different states are clearly observed. (c) The retention of low resistance state and high resistance state under 0.1 V read voltage.

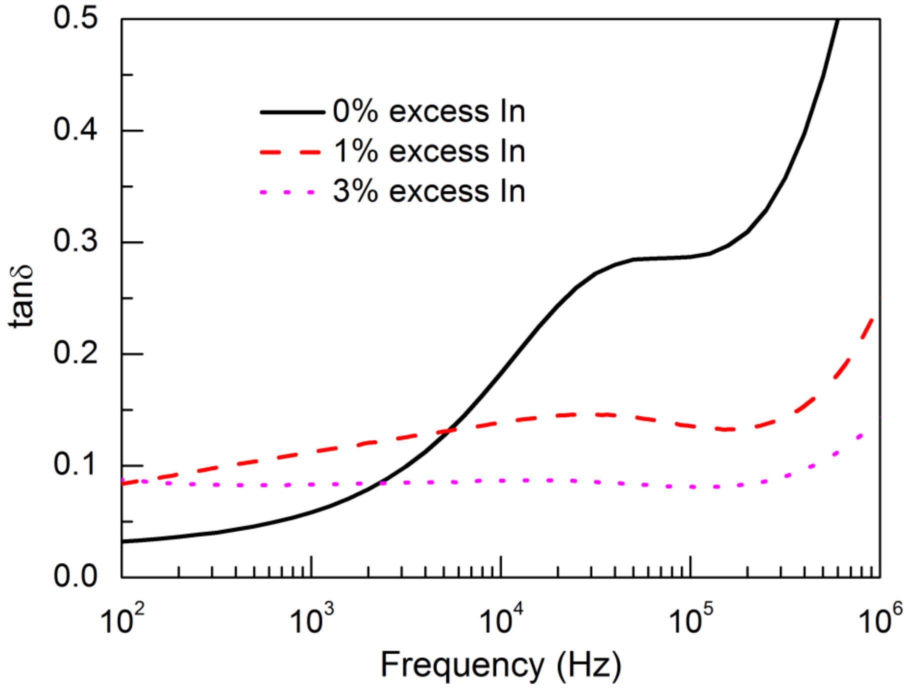

Figure S10. Frequency dependent of dielectric loss of TINO10 ceramics with excess In element, sintering under In-rich atmosphere.
